# Muscle-origin creatinine-cystatin C ratio is an osteoporosis marker in individuals with normal renal function: evidence from observational and Mendelian randomization analysis

**DOI:** 10.3389/fendo.2024.1325320

**Published:** 2024-05-21

**Authors:** Pei He, Yi-Qun Yang, Han Wang, Ya-Qian Zhang, Yu-Ni Gu, Chen-Cheng Hong, Lin Bo, Fei-Yan Deng, Shu-Feng Lei

**Affiliations:** ^1^ Collaborative Innovation Center for Bone and Immunology Between Sihong Hospital and Soochow University, Center for Genetic Epidemiology and Genomics, School of Public Health, Suzhou Medical College of Soochow University, Suzhou, Jiangsu, China; ^2^ Jiangsu Key Laboratory of Preventive and Translational Medicine for Major Chronic Non-communicable Diseases, MOE Key Laboratory of Geriatric Diseases and Immunology, Soochow University, Suzhou, Jiangsu, China; ^3^ Department of Rheumatology, The Second Affiliated Hospital of Soochow University, Suzhou, Jiangsu, China

**Keywords:** creatinine-cystatin C ratio, osteoporosis, Mendelian randomization analyses, linkage disequilibrium score regression, pleiotropic analysis under composite null hypothesis

## Abstract

**Background:**

Creatinine-cystatin C ratio (CCR) has been demonstrated as an objective marker of sarcopenia in clinical conditions but has not been evaluated as an osteoporosis marker in individuals with normal renal function.

**Methods:**

We selected 271,831 participants with normal renal function from UK Biobank cohort. Multivariable linear/logistic regression and Cox proportional hazards model were used to investigate the phenotypic relationship between CCR and osteoporosis in total subjects and gender-stratified subjects. Based on the genome-wide association study (GWAS) data, linkage disequilibrium regression (LDSC) and Mendelian randomization (MR) analysis were performed to reveal the shared genetic correlations and infer the causal effects, respectively.

**Results:**

Amongst total subjects and gender-stratified subjects, serum CCR was positively associated with eBMD after adjusting for potential risk factors (all *P*<0.05). The multivariable logistic regression model showed that the decrease in CCR was associated with a higher risk of osteoporosis/fracture in all models (all *P*<0.05). In the multivariable Cox regression analysis with adjustment for potential confounders, reduced CCR is associated with the incidence of osteoporosis and fracture in both total subjects and gender-stratified subjects (all *P*<0.05). A significant non-linear dose–response was observed between CCR and osteoporosis/fracture risk (*P*_non-linearity_ < 0.05). LDSC found no significant shared genetic effects by them, but PLACO identified 42 pleiotropic SNPs shared by CCR and fracture (P<5×10–^8^). MR analyses indicated the causal effect from CCR to osteoporosis/fracture.

**Conclusions:**

Reduced CCR predicted increased risks of osteoporosis/fracture, and significant causal effects support their associations. These findings indicated that the muscle-origin serum CCR was a potential biomarker to assess the risks of osteoporosis and fracture.

## Introduction

Osteoporosis is a systemic skeletal disorder characterized by decreased bone density, deterioration of bone microarchitecture, and increased bone fragility. This insidious disease significantly heightens susceptibility to fracture, consequently impacting morbidity, mortality, and overall quality of life (1). Osteoporosis is a prevalent condition that primarily affects a substantial number of elderly individuals across various ethnic backgrounds, including both females and males (2). Dual Energy X-ray Absorptiometry (DXA) remains globally recognized as the diagnostic gold standard for assessing bone mineral density (BMD). However, its application in expansive epidemiological studies is hindered by its considerable cost. Therefore, the development of a more specific and sensitive plasma biomarker for early osteoporosis diagnosis has important clinical significance for its prevention and treatment.

Creatinine-cystatin C ratio (CCR) has been recently developed as a sarcopenia index by using two renal functional markers and has received substantial interest as a surrogate measure for muscle mass (3). Cystatin C and creatinine are well-established markers of kidney function. Serum creatinine is a derivative of the skeletal muscle protein and is relatively stable and in proportion to muscle mass. Previous studies have reported that serum creatinine levels can serve as a valuable hematological marker for evaluating muscle mass in individuals with normal renal function (4, 5). In contrast, cystatin C (Cys C), an optimal endogenous marker that accurately reflects variations in glomerular filtration rate, has emerged as a promising surrogate marker for renal function assessment, as it remains unaffected by variations in muscle mass (6). Therefore, when the serum creatinine levels were partially corrected by Cys C, the CCR would be a more accurate measure for residual muscle mass (7). CCR has been demonstrated as an objective marker of sarcopenia in patients with type 2 diabetic patients (8), chronic obstructive pulmonary disease (6), and cancer (9). It is important to note that muscle and bone closely interact through both mechanical forces and the secretion of osteokines and myokines. Reductions in muscle strength (dynapenia), muscle mass (quantity), relative strength (strength per unit of muscle mass), muscle quality (architecture and composition), and/or physical performance (i.e., tasks of functionality) are associated with the age-related health conditions such as osteoporosis. However, no studies have evaluated the muscle-origin CCR as an osteoporosis marker in individuals with normal renal function.

Observational research is an important methodology for gathering evidence on risk factors and causes of health conditions and can offer valuable insights, generating hypotheses that may be unethical or impractical in clinical trials, exploring life-course associations, investigating populations typically excluded from trials, and public health surveillance (10). However, it is essential to acknowledge that confounding represents a significant concern in observational studies (11). Although randomized controlled trials (RCTs) are widely considered as the gold standard for establishing causality in both epidemiological and clinical research, these are time‐consuming, costly, and yields evidence with restricted relevance to practical clinical implementation (12). As an alternative method to RCT, Mendelian randomization (MR) is an effective method to test the etiological hypothesis by effectively applying the statistical data results of existing genome-wide association study (GWAS). This method can effectively avoid the bias in observational epidemiology because of the relative stability of genetic effects and not affected by the environment.

To clarify the utility of the serum Cr/CysC ratio as a promising marker in osteoporosis, this study first investigated the associations between CCR and osteoporosis/fracture based on a large general population cohort from the UK Biobank. Then, linkage disequilibrium score regression (LDSC) and pleiotropic analysis under composite null hypothesis (PLACO) assessed the genetic correlations and specific shared genetic for the phenotypic correlations, respectively. MR analyses were performed to infer the causal effects between CCR and osteoporosis/fracture in European populations.

## Materials and methods

### Human subjects

**Figure 1:**
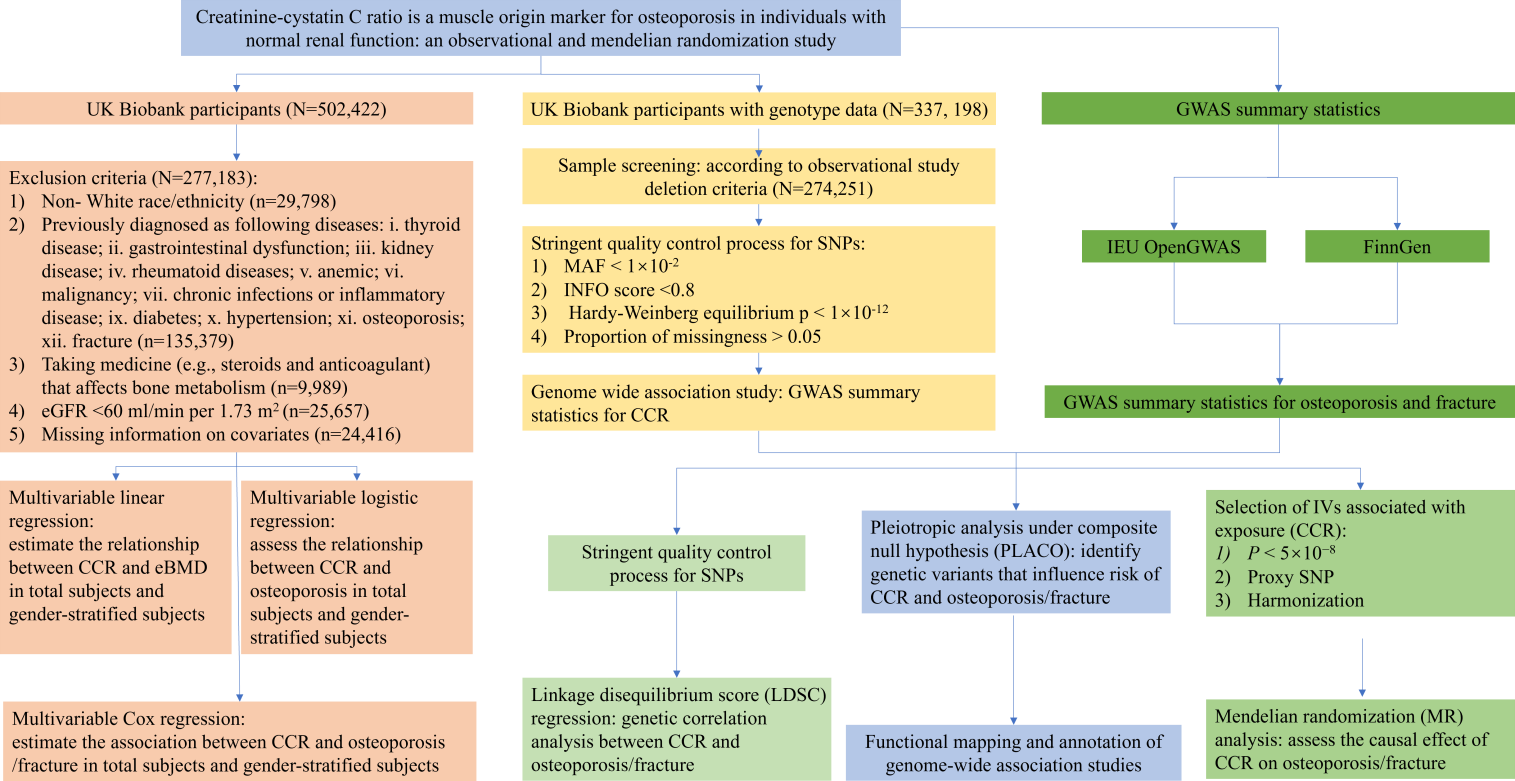
Flowchart of the study.

The UK Biobank is a large-scale prospective cohort that incorporated individual-level data from over 0.5 million participants recruited from 22 assessment centers across the United Kingdom during 2006–2010 (13). The UK Biobank study was approved by the Ethical Committee of North West Multi-center Research (11/NW/0382). Each participant signed a written informed consent document. Necessary individual-level phenotype data of 502,422 subjects were obtained. The individuals were excluded if they: 1) were non- White race/ethnicity (based on UK Biobank Data-Field 21000 “Ethnic background”); 2) were previously diagnosed as following diseases: i. thyroid disease; ii. gastrointestinal dysfunction; iii. kidney disease; iv. rheumatoid diseases; v. anemic; vi. malignancy; vii. chronic infections or inflammatory disease (Data-Field 41271 “Diagnoses - ICD9”, Data-Field 20001 “Cancer code, self-reported”, Data-Field 20002 “No-cancer illness code, self-reported”, Data-Field 20003 “Treatment/medication code”); ix. diabetes; x. hypertension; 3) were taking medicine (e.g., steroids and anticoagulant) that affects bone metabolism (Data-Field 20003 “Treatment/medication code”); 4) with estimated glomerular filtration rate (eGFR) < 60 ml/min per 1.73 m^2^. eGFR was estimated according to the following equations: eGFR (ml/min per 1.73 m^2^) = 186 × Cr^− 1.154^ × age^− 0.203^ ×0.742 (if female) (14). Finally, a total of 271,831 individuals were included for a cross-sectional study. The baseline time was defined as the date the participants first entered the assessment center, between 2006 and 2010 (Data-Field 53 “Date of attending assessment centre”). The details of the UK Biobank Data-Field and Data-Coding for data extraction are described in the **Supplementary Table 1**. The flowchart of the study participants selection and study design are shown in **Figure 1**.

### Genome wide association study summary-statistic data

The summary statistics analyzed were derived from European populations. The summary statistics of osteoporosis and fracture GWAS datasets were downloaded from FinnGen (available at https://r8.finngen.fi/). The Finngen project (available at https://www.finngen.fi) is a large-scale genetic research initiative aimed at exploring the genetic makeup and its relation to various health conditions. The osteoporosis GWAS dataset comprising of ~18 million SNPs is derived from a GWAS study with 5,354 cases and 325,717 controls of European subjects. The dataset for fracture GWAS included ~18 million SNPs involving 262,316 subjects.

The summary statistics of CCR were derived from 337, 198 white British ancestry participants (including 181,063 females and 156,135 males) of the UK Biobank. To be consistent with observational studies, we applied the same sample exclusion criteria as observational studies. A total of 274,251 individuals were included in GWAS analysis, including 149,202 females and 125,049 males. We performed linear mixed model analysis to test the association between autosomal genetic variation and by phenotype using GCTA fastGWA software (15), assuming an additive allelic effect. The fastGWA is a GWAS analysis tool based on a mixed linear model (MLM) in GCTA software. To explain the genetic structure in the cohort, the sparse genome relationship matrix (GRM) was calculated by individuals of independent European descent from the UK Biobank. This method greatly improves the efficiency of analyzing large data set resources such as the UK Biobank. Similar analyses were also conducted in different gender groups. A total of ~92 million variants were generated by imputation, which was performed based on Haplotype Reference Consortium (HRC), UK10K and 1000 Genomes reference panels. We removed SNPs with MAF < 1×10–^2^, INFO score <0.8, Hardy-Weinberg equilibrium p < 1×10–^12^ and proportion of missingness > 0.05. Finally, over 9 million high-quality SNPs remained for further analysis. The following covariates were included as fixed effects: age, gender, genotyping array, assessment center and the first 20 principal components. Manhattan plots were created to visually summarize GWAS results and identify genetic variants significantly associated with CCR. The genome-wide significant threshold of P<5×10–^8^ was used for the GWAS.

### Assessment of exposure, outcome, and covariates

Serum cystatin C (Data-Field 30720 “Cystatin C”) and creatinine (Data-Field 30700 “Creatinine”) concentrations were measured at baseline. Serum cystatin C concentration was measured using a latex‐enhanced immuno‐turbidimetric assay by Siemens (Erlangen, Germany) on the Siemens Advia 1800, with an interassay coefficient of variation of 1.1% (16). Serum creatinine concentration was measured using an enzyme‐based assay by Beckman Coulter (High Wycombe, United Kingdom) on the Beckman Coulter AU5800, with a coefficient of variation of 2.0% (16). The details of sample collection and processing were previously described (17).

BMD derived from calcaneus ultrasound was measured by the Sahara Clinical Bone Sonometer (Hologic Corporation, Bedford, MA, USA). T-score derived from quantitative ultrasound (QUS) of the heel estimated BMD (eBMD) [Data-Field 3148 “Heel bone mineral density (BMD)”, Data-Field 3084 “Heel bone mineral density (BMD), manual entry”, Data-Field 4105 “Heel bone mineral density (BMD) (left)”, Data-Field 77 “Heel bone ultrasound T-score, manual entry”, Data-Field 78 “Heel bone mineral density (BMD) T-score, automated”, Data-Field 4106 “Heel bone mineral density (BMD) T-score, automated (left)”] was used to diagnose osteoporosis. With reference to the current WHO recommendations, we defined osteoporosis as T score -2.5 ≤SD, and non-osteoporosis as T > -2.5. The ICD-10 code used to diagnose diseases was **Supplementary Table 1**.

Based on previous studies, variables that could influence the correlation between CCR and osteoporosis/fracture risk were considered covariates in our analysis. These variables include age (Data-Field 21003 “Age at recruitment”), body mass index (BMI) [Data-Field 21001 “ Body mass index (BMI)”], height (Data-Field 50 “Standing height”), assessment centre (Data-Field 54 “UK Biobank assessment centre”), smoking status (Data-Field 20116 “Smoking status”), alcohol consumption (Data-Field 20117 “Drinking status”), medication treatments, and physical activity (Data-Field 874 “Duration of walks”, Data-Field 884 “Number of days/week of moderate physical activity 10+ minutes”, Data-Field 894 “Duration of moderate activity”, Data-Field 904 “ Number of days/week of vigorous physical activity 10+ minutes”, Data-Field 914 “ Duration of vigorous activity”) for both male and female, and hormone replacement therapy (HRT) [Data-Field 2814 “Ever used hormone-replacement therapy (HRT)”] and menopausal (Data-Field 2724 “Menopausal status”) for female. Physical activity duration was estimated to be the sum of MET minutes per week of walking and engaging in moderate and vigorous activity. A MET is estimated according to the energy cost of a given activity divided by resting energy expenditure (18). One minute of walking, moderate and vigorous activity were 3.3 METS, 4 METS and 8 METS, respectively. The detail of the UK Biobank Data-Field and Data-Coding for data extraction is described in the **Supplementary Table 1**.

### Observational studies

We conducted a cross-sectional study to investigate the association between CCR and eBMD/osteoporosis by using individual-level data from 271,831 individuals. A multivariable linear regression was performed to estimate the relationship between CCR and eBMD in total subjects and gender-stratified subjects, controlling for potential confounders. We applied a multivariable logistic regression to assess the relationship between CCR and osteoporosis in total subjects and gender-stratified subjects, controlling for potential confounders. Here the confounders were set in three ways: model 1 [including age, height, weight, BMI, assessment centre, HRT (only in female), menopausal status (only in female), smoking status, and drinking status], model 2 (model 1+ eGFR), model 3 (model1+ regular physical activity).

The follow-up time referred to the period from baseline enrollment to the first diagnosis of osteoporosis/fracture (Data-Field 41280 “Date of first in-patient diagnosis - ICD10”), the first registration of osteoporosis/fracture or loss (Data-Field 191 “Date loss to follow-up”), or death (Data-Field 40000 “Date of death”), or end of follow-up (31 May 2022). Cox proportional hazard models was performed to estimate the association between CCR and osteoporosis/fracture (including all fractur) in total subjects and gender-stratified subjects with the adjusted for age, height, weight, BMI, assessment centre, HRT (only in female), menopausal status (only in female), smoking status and drinking status in model 1, and model 2 adjusted for additional eGFR, and model 3 adjusted for additional regular physical activity based on model 2. Hazard ratios (HRs) and 95% confidence intervals (CIs) were assessed. Likelihood ratio, Wald test and Score (logrank) tests were used to determine statistical significance. The association between cystatin CCR and osteoporosis/fracture risk were further investigated by using restricted cubic spline models fitted for Cox proportional hazards models with 4 knots at the 5th, 35th, 65th, and 95th percentiles. The likelihood ratio test was used to calculate P-value for non-linearity. R Software (version 4.2.0) was used for data management and statistical analyses.

### Genetic correlation

Linkage disequilibrium score (LDSC) regression was performed to infer SNP-based heritability and genetic correlation estimates from GWAS summary data by using the deviation of the observed χ^2^ test statistic for an SNP from its expected value under the null hypothesis of no association (19). LDSC was conducted using the 1000 Genomes project as a reference panel. For SNPs, stringent quality control was implemented, by removing all non-biallelic allele SNPs, SNPs with strand-ambiguous alleles (A/T, C/G allele SNPs), SNPs with minor allele frequency (MAF) < 1%, SNPs lacking rs numbering, duplicate rs IDs, and SNPs that were not presented or whose alleles did not match phase 3 of the 1000 Genomes Project.

### Pleiotropic enrichment analysis

We used a statistical approach pleiotropic analysis under composite null hypothesis (PLACO) that uses GWAS summary statistics to identify genetic variants that influence risk of CCR and osteoporosis/fracture (20). This approach has improved performance over other existing methods, both Bayesian and frequentist, in most scenarios. PLACO employed a null hypothesis testing approach utilizing the product of Z statistics derived from the SNP data in the two summary statistics. It subsequently constructed a null distribution for the test statistic, adopting a mixture distribution framework that accommodated scenarios where subsets of SNPs were associated exclusively with either one or none of the phenotypes under investigation. This approach allowed for a comprehensive evaluation of the genetic associations across multiple phenotypes while considering the potential complexity of the underlying genetic architecture. The principle and algorithm of PLACO were well-described in elsewhere (20). To minimize the occurrence of false-positive findings, we implemented a rigorous Bonferroni correction method, setting the significance threshold at a P-value < 0.05/number of SNPs in each analysis. Functional mapping and annotation of genome-wide association studies (FUMA, available at: https://fuma.ctglab.nl/) was adopted to assess the biological function of pleiotropic (21). The threshold of r^2^ defining independent significant SNPs was set to 0.2, and the maximum distance of 500 kb was used to merge linkage disequilibrium (LD) blocks into a locus. The identified genomic locus was subsequently mapped to proximal genes, and a comprehensive set of pathway enrichment analyses was employed to elucidate the functional implications of the mapped genes, leveraging the Molecular Signature Database (MSigDB).

### Causal association analysis

The potential causal effect of a risk factor (e.g., CCR) on the outcome (osteoporosis, fracture) was assessed by Mendelian randomization (MR) analysis (22). Here, three MR methods including the inverse-variance weighted method (IVW), weighted median regression, and MR-Egger regression were used in this study. Cochran Q statistic was used to assess the heterogeneity. Leave-one-out sensitivity analysis was performed to identify single SNP with potential impact. MR-Egger intercept and the Mendelian Randomization Pleiotropy RESidual Sum and Outlier (MR-PRESSO) global test were used to assess the potential horizontal pleiotropy between IVs and outcome (23). Summary statistics for CCR SNPs with p < 5×10^−8^ were extracted from the GWAS dataset. Reducing clusters of SNPs in LD to a single SNP eliminated any dependency between SNPs. Proxy SNP which was in high LD (r^2^ > 0.8) with the SNP of interest was included only for the osteoporosis/fracture dataset when target SNP was not available in osteoporosis/fracture dataset. Harmonization ensured that the effect of an instrumental SNP on the risk factor and the effect of the SNP on outcome corresponded to the same allele. All the analyses were performed using the R package “TwoSampleMR”. LD between chosen SNPs was estimated based upon the genotype data of European samples from the 1000 Genomes project.

## Results

The baseline characteristics of the study subjects are shown in **Supplementary Table 2**. The cross-sectional study included 277,183 participants, including 150,869 women and 126,314 men. The mean (SD) age was 55.50 (8.11) years, and the mean (SD) BMI was 26.78 (447) kg/m^2^. Compared with female subjects, the male subjects had higher CCR (9.98 *v.s.* 8.51) and eBMD (0.58 *v.s.* 0.52).

In both total subjects and gender-stratified subjects, CCR was significantly associated with eBMD by using multivariable linear regression after adjusted for potential risk factors (in Model 1, model 2 and model 3) (*p*<0.05) (**Supplementary Table 3**). In addition, the effects of CCR on osteoporosis were estimated using the multivariable logistic regression model. We observed that the decrease of CCR was associated with higher risk of osteoporosis when diagnosed by using eBMD in all models (**Supplementary Table 4**).

**Figure 2:**
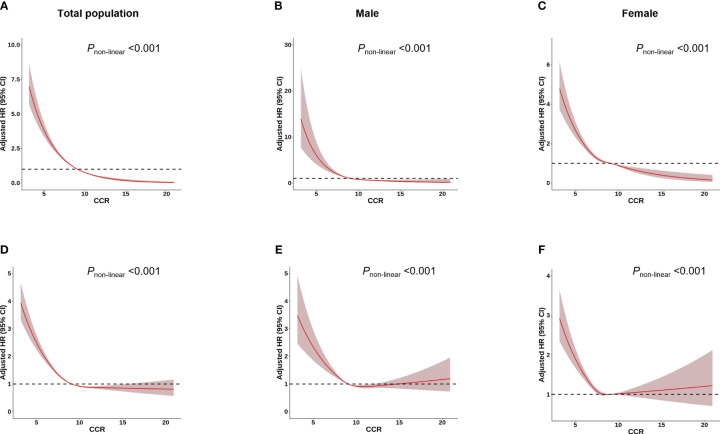
Association between CCR and osteoporosis/fracture risk using a restricted cubic spline regression Model. Results were adjusted for age, height, weight, BMI, assessment centre, HRT (only in female), menopausal status (only in female), smoking status, drinking status, regular physical activity and estimated glomerular filtration rate. The red shadow represents the 95% confidence intervals for the spline mode. **(A-C)** Analysis of the shape of the relationship between CCR and osteoporosis risk. **(D-F)** Analysis of the shape of the relationship between CCR and fracture risk.

Among 277,183 participants, 7,177 incident cases of osteoporosis were recorded during a median follow-up of 8.45 years (**Table 1**). In the multivariable Cox regression analysis with adjustment for age, height, weight, BMI, assessment center, HRT (only in female), menopausal status (only in female), smoking status, drinking status, eGFR and physical activity, we observed that CCR were associated with osteoporosis. A negative association was observed between CCR and the risk of osteoporosis in both total subjects and gender-stratified subjects in all models [Model 1 (Total: HR=0.75, 95% CI: 0.73–0.76; Male: HR=0.72, 95%CI: 0.68–0.75; Female: HR=0.82, 95%CI: 0.80–0.84), Model 2 (Total: HR=0.73, 95% CI: 0.71–0.74; Male: HR=0.74, 95%CI: 0.70–0.78; Female: HR=0.83, 95%CI: 0.82–0.86) and Model 3 (Total: HR=0.73, 95% CI: 0.71–0.75; Male: HR=0.74, 95%CI: 0.70–0.79; Female: HR=0.83, 95%CI: 0.81–0.86)] (**Table 2**). Individuals with higher CCR had a lower risk of osteoporosis than those with lower CCR (**Table 3**). A significant non-linear dose–response was observed between CCR and osteoporosis risk (*P*_non-linearity_ < 0.05, **Figure 2**).

**Table 1:** Comparison of basic characteristics between the disease group and the non-disease.

Characteristics	Incident osteoporosis						Incident fracture					
	Yes			No			Yes			No		
	Female (n=6,068)	Male (n=1,109)	Total (n=7,177)	Female (n=144,801)	Male (n=125,205)	Total (n=270,006)	Female (n=9,227)	Male (n=5,457)	Total (n=14,684)	Female (n=141,642)	Male (n=120,857)	Total (n=262,499)
Age at baseline [y (mean ± SD)]	60.98 ± 6.14	61.06 ± 6.53	60.99 ± 6.21	54.99 ± 7.98	55.78 ± 8.23	55.36 ± 8.11	58.16 ± 7.58	56.43 ± 8.47	57.52 ± 7.96	55.04 ± 8.00	55.80 ± 8.22	55.39 ± 8.11
Height [cm (mean ± SD)]	161.49 ± 6.41	173.92 ± 6.91	163.41 ± 7.89	163.08 ± 6.19	176.25 ± 6.71	169.19 ± 9.20	163.01 ± 6.37	176.21 ± 7.07	167.91 ± 9.21	163.02 ± 6.20	176.23 ± 6.71	169.10 ± 9.21
Weight [kg (mean ± SD)]	65.86 ± 12.35	80.34 ± 15.30	68.10 ± 13.86	70.19 ± 13.18	84.87 ± 13.52	76.99 ± 15.22	69.76 ± 13.24	84.13 ± 14.14	75.10 ± 15.26	70.03 ± 13.18	84.86 ± 13.52	76.86 ± 15.25
Follow-up time [y, median (IQR)]	8.43 (4.60)	8.55 (4.79)	8.45 (4.62)	13.35 (1.37)	13.30 (1.42)	13.32 (1.39)	13.16 (1.80)	13.21 (1.66)	13.16 (1.80)	13.30 (1.41)	13.29 (1.42)	13.29 (1.42)
Body mass index [kg=m2 (mean ± SD)]	25.26 ± 4.59	26.53 ± 4.59	25.45 ± 4.62	26.40 ± 4.80	27.30 ± 3.97	26.482 ± 4.46	26.26 ± 4.84	27.08 ± 4.18	26.57 ± 4.62	26.36 ± 4.80	27.30 ± 3.97	26.79 ± 4.46
Creatinine [umol/L (mean ± SD)]	61.89 ± 9.51	76.86 ± 12.48	64.20 ± 11.39	63.31 ± 9.00	80.19 ± 11.17	71.14 ± 13.12	62.67 ± 9.29	78.66 ± 11.81	68.62 ± 12.88	63.29 ± 9.00	80.23 ± 11.15	71.09 ± 13.13
Cystatin C [mg/L (mean ± SD)]	0.89 ± 0.14	0.96 ± 0.15	0.90 ± 0.14	0.85 ± 0.13	0.92 ± 0.12	0.88 ± 0.13	0.87 ± 0.14	0.92 ± 0.13	0.89 ± 0.14	0.85 ± 0.13	0.92 ± 0.12	0.88 ± 0.13
Creatinine to Cystatin C ratio	7.99 ± 1.25	9.18 ± 1.47	8.17 ± 1.36	8.53 ± 1.34	9.99 ± 1.34	9.20 ± 1.58	8.24 ± 1.36	9.76 ± 1.54	8.81 ± 1.60	8.53 ± 1.34	9.99 ± 1.47	9.20 ± 1.58
Physical activity [MET-min/wk (mean ± SD)]	1731.46 ± 2685.51	1914.92 ± 3463.74	1759.81 ± 2820.33	1655.99 ± 2368.60	2193.87 ± 3450.21	1905.41 ± 2932.69	2009.83 ± 3123.36	2500.58 ± 3813.68	2009.83 ± 3123.36	1655.08 ± 2368.13	2177.47 ± 3432.47	1895.59 ± 2918.61
eGFR (ml/min per 1.73 m^2^)	93.31 ± 17.38	98.43 ± 20.57	94.1 ± 18.01	92.53 ± 15.93	94.57 ± 16.12	93.48 ± 16.05	94.28 ± 17.42	96.88 ± 17.94	94.28 ± 17.42	92.55 ± 15.93	94.51 ± 16.08	93.45 ± 16.03
eBMD	0.44 ± 0.11	0.50 ± 0.13	0.44 ± 0.11	0.52 ± 0.12	0.58 ± 0.14	0.55 ± 0.13	0.50 ± 0.13	0.54 ± 0.14	0.50 ± 0.13	0.52 ± 0.12	0.58 ± 0.14	0.55 ± 0.13
Smoking status (%)												
Never	3363 (55.42)	438 (39.50)	3801 (52.96)	86510 (59.75)	150903 (55.89)	64393 (51.43)	5297 (57.41)	2649 (48.54)	7946 (54.11)	84576 (59.71)	62182 (51.45)	146758 (55.91)
Previous	2070 (34.11)	474 (42.74)	2544 (35.45)	45051 (31.11)	90525 (33.53)	45474 (36.32)	3008 (32.60)	1983 (36.34)	4991 (33.99)	44113 (31.14)	43965 (36.38)	88078 (33.55)
Current	604 (9.96)	189 (17.04)	793 (11.05)	12843 (8.87)	27842 (10.31)	14999 (11.98)	881 (9.55)	808 (14.81)	1689 (11.50)	12566 (8.87)	14380 (11.90)	26946 (10.27)
Prefer not to answer	31 (0.51)	8 (0.72)	39 (0.54)	397 (0.27)	736 (0.27)	339 (0.27)	41 (0.44)	17 (0.31)	58 (0.40)	387 (0.28)	330 (0.27)	717 (0.27)
Drinking status (%)												
Never	370 (6.10)	28 (2.53)	398 (5.55)	5431 (3.75)	7478 (2.77)	2047 (1.63)	181 (2.52)	93 (1.70)	517 (3.52)	5377 (3.80)	1982 (1.64)	7359 (2.80)
Previous	299 (4.93)	71 6.40)	370 (5.16)	4457 (3.08)	8062 (2.99)	3605 (2.88)	239 (3.33)	185 (3.39)	517 (3.52)	4424 (3.12)	3491 (2.89)	7915 (3.02)
Current	5389 (88.81)	1007 (90.80)	6396 (89.11)	134820 (93.11)	254307 (94.18)	119487 (95.44)	6747 (94.02)	5172 (94.78)	13629 (92.82)	131752 (93.02)	115322 (95.42)	247074 (94.12)
Prefer not to answer	10 (0.16)	3 (0.27)	13 (0.18)	93 (0.06)	159 (0.06)	66 (0.05)	9 (0.13)	7 (0.13)	21 (0.14)	89 (0.06)	62 (0.05)	151 (0.06)

**Table 2:** Association of CCR and osteoporosis/fracture.

Group	Model	Osteoporosis		Fracture	
		HR (95% CI)	P-value	HR (95% CI)	P-value
Male and female	1	0.7472 (0.7331, 0.7616)	<2.00E-16	0.8810 (0.8703, 0.8919)	<2.00E-16
	2	0.7265 (0.7105, 0.7429)	<2.00E-16	0.8861 (0.8735, 0.8988)	<2.00E-16
	3	0.7286 (0.7125, 0.7450)	<2.00E-16	0.8843 (0.8718, 0.8970)	<2.00E-16
Male	1	0.7159 (0.6833, 0.7501)	<2.00E-16	0.8996 (0.8818, 0.9177)	<2.00E-16
	2	0.7423 (0.7030, 0.7838)	<2.00E-16	0.9403 (0.9181, 0.9631)	4.59E-07
	3	0.7437 (0.7044, 0.7851)	<2.00E-16	0.9400 (0.9178, 0.9628)	4.25E-07
female	1	0.8221 (0.8038, 0.8408)	<2.00E-16	0.9217 (0.9057, 0.9381)	<2.00E-16
	2	0.8318 (0.8088, 0.8554)	<2.00E-16	0.9448 (0.9243, 0.9657)	3.83E-07
	3	0.8325 (0.8095, 0.8561)	<2.00E-16	0.9446 (0.9241, 0.9655)	3.50E-07

model 1 [including age, height, weight, BMI, assessment centre, HRT (only in female), menopausal status (only in female), smoking status and drinking status], model 2 (model 1+ eGFR), model 3 (model 2+ regular physical activity).

**Table 3:** Multivariable Cox regression analysis for risk of osteoporosis/fracture associated with CCR.

			Model 1		Model 2		Model 3	
	No.	Incident cases	HR (95% CI)	P-value	HR (95% CI)	P-value	HR (95% CI)	P-value
Osteoporosis								
Total								
Q1	69335	3552	1.00 (REF)		1.00 (REF)		1.00 (REF)	
Q2	69288	1982	0.7108 (0.6720, 0.7518)	< 2E-16	0.7109 (0.6707, 0.7536)	< 2E-16	0.7137 (0.6732, 0.7565)	< 2E-16
Q3	69270	1098	0.5062 (0.4716, 0.5434)	< 2E-16	0.5064 (0.4699, 0.5457)	< 2E-16	0.5101 (0.4733, 0.5497)	< 2E-16
Q4	69290	545	0.337 (0.3063, 0.3707)	< 2E-16	0.3371 (0.3047, 0.3731)	< 2E-16	0.3402 (0.3074, 0.3765)	< 2E-16
Male								
Q1	31588	509	1.00 (REF)		1.00 (REF)		1.00 (REF)	
Q2	31587	268	0.5782 (0.4979, 0.6714)	6.63E-13	0.6341 (0.5442, 0.7388)	5.18E-09	0.6358 (0.5457, 0.7409)	6.41E-09
Q3	31590	203	0.4897 (0.4147, 0.5782)	< 2E-16	0.5647 (0.4750, 0.6712)	9.09E-11	0.5650 (0.4753, 0.6715)	9.33E-11
Q4	31549	129	0.3617 (0.2964, 0.4414)	< 2E-16	0.4450 (0.3607, 0.5489)	4.03E-14	0.4449 (0.3607, 0.5487)	3.78E-14
Female								
Q1	37744	2333	1.00 (REF)		1.00 (REF)		1.00 (REF)	
Q2	37727	1653	0.7763 (0.7284, 0.8273)	6.57E-15	0.8114 (0.7589, 0.8675)	9.10E-10	0.8123 (0.7597, 0.8685)	1.12E-09
Q3	37704	1265	0.7000 (0.6525, 0.7509)	< 2E-16	0.7515 (0.6954, 0.8120)	4.91E-13	0.7525 (0.6964, 0.8132)	6.38E-13
Q4	37694	817	0.5668 (0.5213, 0.6162)	< 2E-16	0.6287 (0.5711, 0.6921)	< 2E-16	0.6302 (0.5724, 0.6937)	< 2E-16
Fracture								
Total								
Q1	69334	4998	1.00 (REF)		1.00 (REF)		1.00 (REF)	
Q2	69286	3762	0.7876 (0.7542, 0.8224)	< 2E-16	0.8034 (0.7684, 0.8401)	< 2E-16	0.8013 (0.7663, 0.8379)	< 2E-16
Q3	69273	3162	0.6872 (0.6554, 0.7204)	< 2E-16	0.7090 (0.6743, 0.7455)	< 2E-16	0.7058 (0.6712, 0.7421)	< 2E-16
Q4	69290	2762	0.6342 (0.6023, 0.6678)	< 2E-16	0.6651 (0.6279, 0.7046)	< 2E-16	0.6610 (0.6240, 0.7003)	< 2E-16
Male								
Q1	31588	1682	1.00 (REF)		1.00 (REF)		1.00 (REF)	
Q2	31587	1353	0.7936 (0.7382, 0.8531)	3.77E-10	0.8540 (0.7925, 0.9204)	3.57E-05	0.8534 (0.7919, 0.9197)	3.30E-05
Q3	31590	1248	0.7360 (0.6827, 0.7935)	1.34E-15	0.8263 (0.7623, 0.8957)	3.50E-06	0.8274 (0.7632, 0.8969)	4.15E-06
Q4	31549	1174	0.7023 (0.6494, 0.7595)	< 2E-16	0.8341 (0.7630, 0.9118)	6.57E-05	0.8342 (0.7629, 0.9120)	6.82E-05
Female								
Q1	37744	2999	1.00 (REF)		1.00 (REF)		1.00 (REF)	
Q2	37727	2363	0.8380 (0.7934, 0.8851)	2.37E-10	0.8728 (0.8243, 0.9242)	3.19E-06	0.8726 (0.8240, 0.9240)	3.05E-06
Q3	37704	2035	0.7889 (0.7442, 0.8363)	1.66E-15	0.8425 (0.7900, 0.8984)	1.75E-07	0.8422 (0.7898, 0.8982)	1.67E-07
Q4	37694	1830	0.7999 (0.7512, 0.8516)	3.02E-12	0.8820 (0.8187, 0.9501)	9.39E-04	0.8814 (0.8182, 0.9496)	8.90E-04

HR, hazard ratio; REF, reference.

model 1 [including age, height, weight, BMI, assessment centre, HRT (only in female), menopausal status (only in female), smoking status and drinking status], model 2 (model 1+ eGFR), model 3 (model 2+ regular physical activity).

Over a median follow-up of 13.16 years, 14,684 of the 277,183 participants suffered fracture (**Table 1**). The multivariable Cox regression analysis indicated that CCR was associated with fracture (**Table 2**). A negative association was observed between CCR and the risk of fracture in both total subjects and gender-stratified subjects in all models [Model 1 (Total: HR=0.88, 95% CI: 0.87–0.89; Male: HR=0.90, 95%CI: 0.88–0.92; Female: HR=0.92, 95%CI: 0.91–0.94) and Model 2 (Total: HR=0.89, 95% CI: 0.87–0.90; Male: HR=0.94, 95%CI: 0.92–0.96; Female: HR=0.94, 95%CI: 0.92–0.97); Model 3 (Total: HR=0.88, 95% CI: 0.87–0.90; Male: HR=0.94, 95%CI: 0.92–0.96; Female: HR=0.94, 95%CI: 0.92–0.97)] (**Table 2**). Individuals with higher CCR had a lower risk of fracture than those with lower CCR (**Table 3**). A significant non-linear dose–response was observed between CCR and fracture risk (*P*_non-linearity_ < 0.05, **Figure 2**).

**Figure 3:**
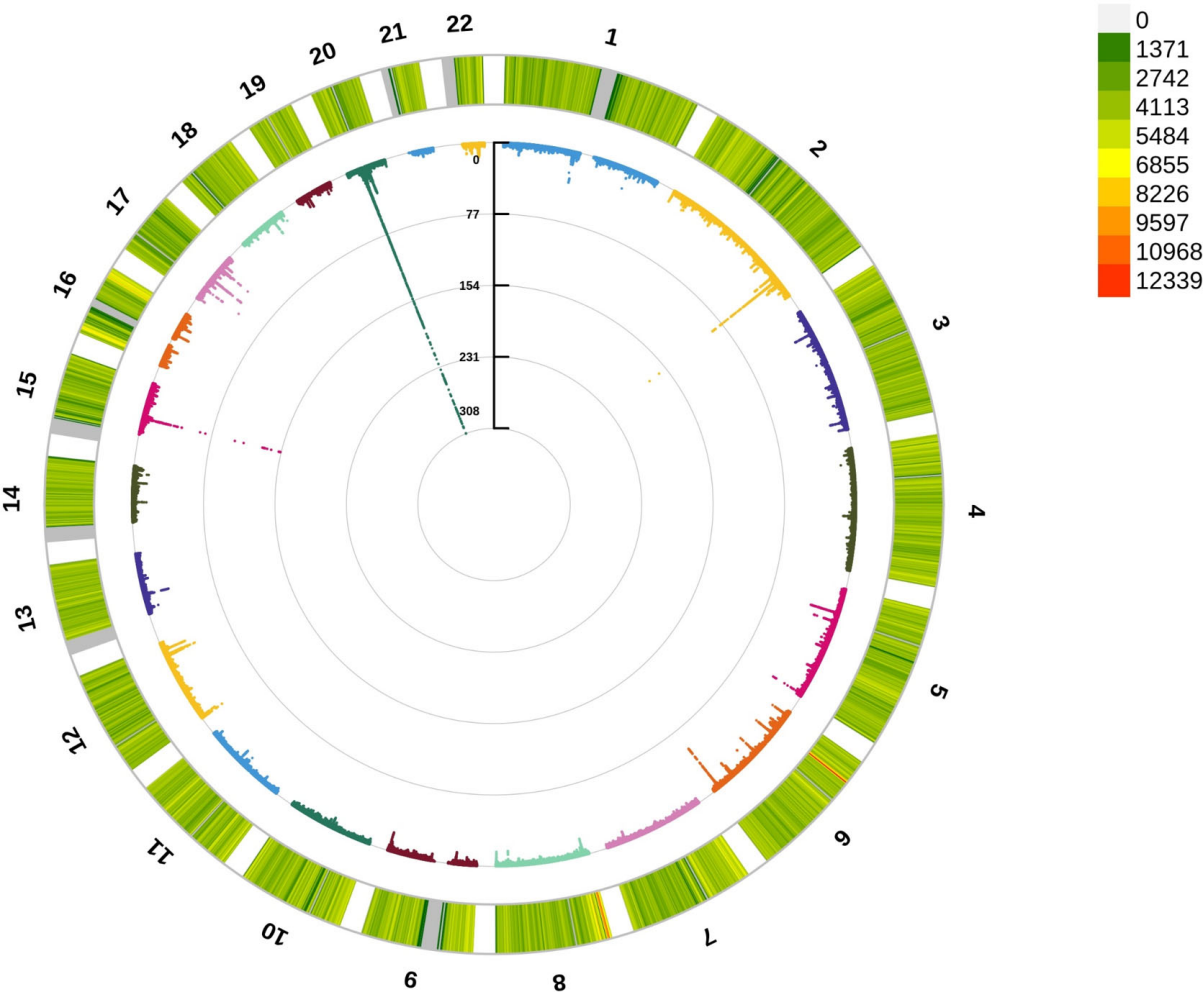
Circle Manhattan plot of the CCR GWAS results. The red line marking the significance of 5×10–^8^.

To detect the underlying mechanism for the above significant phenotypic correlations between CCR and osteoporosis, we first performed a GWAS analysis for CCR. A total of 274,251 white British ancestry participants (including 149,202 females and 125,049 males) included in a CCR GWAS (**Supplementary Table 5**). We analyzed 110,639,001 autosomal SNPs for their association with CCR. About 13,992 SNPs had results passing the significance threshold of 5×10–^8^ in the GWAS of CCR (**Figure 3**; **Supplementary Table 6**). The 13,992 SNPs associated with CCR were in 22 genomic risk loci, corresponding to 488 lead SNPs (**Supplementary Table 7**). These lead SNPs were mapped to 811 genes, which were enriched in multiple cellular components, including cytosol, cytoplasm, nucleoplasm, membrane, secretory granule membrane, apical plasma membrane and perinuclear region of cytoplasm. They executed molecular functions, including protein binding, cysteine-type endopeptidase inhibitor activity, quaternary ammonium group transmembrane transporter activity, phosphotyrosine binding, xenobiotic transporter activity and organic cation transmembrane transporter activity, and were involved in negative regulation of peptidase activity and organic cation transport (**Supplementary Table 8**).

Based on the CCR GWAS data, we performed LDSC analysis to detect the genetic correlation between CCR and osteoporosis/fracture, but no significant genetic correlations were detected between them (**Supplementary Table 9**). However, further PLACO analyses identified significant 119 pleiotropic SNPs shared by CCR and osteoporosis (Bonferroni adjusted P<0.05) (**Supplementary Table 10**). Among these pleiotropic SNPs, 57.1% (68 SNPs) were intronic, 13.4% were nearby 3’ or 5’ terminal of genes. FUMA showed the 119 pleiotropic SNPs were in 9 genomic risk loci, corresponding to 9 lead SNPs (**Supplementary Table 11**). In addition, forty-two pleiotropic SNP shared by CCR and fracture were detected using PLACO method (Bonferroni adjusted P<0.05) (**Supplementary Table 10**). These pleiotropic SNPs were located within 4 genomic risk loci, corresponding to 4 lead SNPs (**Supplementary Table 11**). The lead SNPs were mapped to 20 genes. GO analyses indicated that these genes were significantly enriched in biological processes of respiratory electron transport chain and cellular response to extracellular stimulus. The molecular functions for these genes were mainly involved in polyubiquitin modification-dependent protein binding (**Supplementary Figure 1**).

To further detect the underlying mechanism for the above significant phenotypic correlations between CCR and osteoporosis, we performed Mendelian randomization analysis to infer the causal effects between them by using our CCR GWAS data and publicly available summary data for osteoporosis. One hundred and nineteen independent SNPs were selected as instrumental variables for the CCR for the MR analysis (**Supplementary Table 12**). Cochran Q statistics and MR PRESSO all indicated the absence of a directional pleiotropic effect in MR analysis assessing the effects of CCR on fracture. Cochran Q statistics showed that there was heterogeneity in the effect of CCR estimated by SNPs on osteoporosis (**Supplementary Table 13**). Therefore, causal variant effects were examined with the IVW method with multiplicative random effects between CCR and osteoporosis. IVW MR results showed that genetically predicted CCR was associated with both osteoporosis (beta, -0.016, 95% CI: -0.028 to -0.005) and fracture (beta, -0.024, 95% CI: -0.003, -0.045) (**Table 4**). MR-Egger intercept suggested that there was horizontal pleiotropy in MR analysis of CCR and osteoporosis. The MR-PRESSO distortion test showed an insignificant association between CCR and osteoporosis (p>0.05). However, the association between CCR and osteoporosis/fracture became insignificant with the MR-Egger and weighted median methods.

**Table 4:** Mendelian randomization analyses of CCR with osteoporosis/fracture.

Outcome	Exposure	Number of SNPs	IVW (multiplicative random effects)		Weighted median		MR-Egger			
			β (95% CI)	P-value	β (95% CI)	P-value	β (95% CI)	P-value	Intercept (95%CI)	P-value
Osteoporosis	CCR	119	-0.016 (-0.028, -0.005)	5.81E-03	-0.006 (-0.022, 0.010)	0.46	0.007 (-0.013, 0.026)	0.50	-0.011 (-0.019, -0.00)	5.34E-03
Fracture	CCR	137	-0.024 (-0.003, -0.045)	0.02	-0.017 (-0.048, 0.015)	0.31	-0.007 (-0.043, 0.029)	0.71	-0.008E-03 (-0.023, 0.006)	0.25

## Discussion

The study first systematically assessed the relationship between CCR and osteoporosis in a large population-based cohort by integrating multiple methods including correlation analysis, LDSC, PLACO, and MR (**Supplementary Figure 2**). Based on the largescale dataset of the UK Biobank, we conducted a prospective cohort study to investigate the association of CCR and risk of incident osteoporosis/fracture. By analyzing hundreds of thousand subjects, we found decreased CCR at baseline was associated with increased risk of incident osteoporosis/fracture during the follow-up. MR analyses confirmed the causal effects between them. Then, common genetic variants behind the CCR and osteoporosis/fracture were analyzed by using PLACO. These findings suggest that the CCR, being a rapidly measurable and widely available biomarker, holds promise as a potential indicator for predicting the risk of incident osteoporosis/fracture. These results contribute to a better understanding of the pathogenesis of osteoporosis/fracture and may have implications for the development of preventive and diagnostic strategies in the field of bone health.

Previous studies have provided evidence of significant associations between reduced ratios of serum CCR and diminished muscle mass, as well as adverse clinical outcomes in multiple disease conditions (24–27). However, there has been limited investigation into the potential relationship between CCR and bone properties. A small-scale study in postmenopausal women in Japan (n=60) showed that the CCR was positively correlated with BMD (28). Furthermore, the relationship between CCR and speed of sound (SOS) at calcaneal bone was evaluated in a general population-based cohort of Japan, and it indicated that CCR was positively associated with SOS in both female and male (29). Building upon this existing research, the current study sought to examine the association between the CCR and estimated bone mineral density (eBMD) while also investigating its potential relationship with osteoporosis and fracture risk. The present study suggested a positive association between CCR and eBMD, and a negative association between CCR and osteoporosis/fracture. In light of the observed positive correlation between the CCR and bone mineral density, as well as the negative association between the CCR and osteoporosis, several plausible biological mechanisms warrant considered. First, the CCR may be linked to muscle mass and function. The musculoskeletal system is closely intertwined with the muscular system, and muscle movement and strength are crucial for maintaining healthy bone density and structure (30, 31). The CCR can be used to reflect systemic muscle mass and muscle function (32). Reduced muscle mass or sarcopenia is a well-known risk factor for osteoporosis and fracture (33). Higher muscle mass and function may be associated with higher bone mineral density, while a reduced CCR may reflect muscle loss or diminished function, correlating with decreased bone mineral density. Second, the CCR may be related to inflammation and the status of chronic diseases. Chronic inflammation and certain chronic diseases, such as chronic kidney disease, have been implicated in the development of osteoporosis (34). An elevated CCR may indicate a milder inflammatory state or slower progression of chronic diseases, thereby reducing the risk of osteoporosis. Lastly, the CCR may reflect the metabolic state of the body. Metabolic disturbances have been consistently associated with reduced bone density and the development of osteoporosis (35). A higher CCR may be indicative of better metabolic health, potentially contributing to the maintenance of bone density. It is imperative to emphasize that further in-depth research is warranted to elucidate these potential mechanisms with greater precision and comprehensiveness.

Observational studies observed that the CCR is positively correlated with eBMD while showing a negative correlation with osteoporosis or fractures. These findings suggested that higher CCR may be indicative of improve bone health, potentially mitigating the risk of osteoporosis. Nevertheless, observational studies cannot establish causality and only provide clues about associations. To enhance our understanding and establish a potential causal link, Mendelian randomization analysis was employed, which yielded results supporting the notion of a causal relationship between the CCR and osteoporosis. This analytical approach helped mitigate the influence of certain confounding factors. However, the results of LDSC analysis showed no evidence of genetic correlation between the CCR and osteoporosis. This may suggest that the impact of the CCR on bone mineral density is primarily driven by environmental factors rather than genetic inheritance. Nevertheless, it is important to note that genetic correlation analysis has inherent limitations and may not completely negate the potential involvement of other contributing factors. The relationship between the CCR and osteoporosis is a multifaceted issue involving genetics, environment, and potential causality. Furthermore, PLASCO analysis revealed the presence of pleiotropic SNPs in the association between the CCR and osteoporosis. This observation suggested that different genetic variations may have varying impacts on this association in different populations. This further emphasized the complexity of osteoporosis, where individual genetic backgrounds may play divergent roles in shaping this relationship. It is imperative to recognize that the results obtained through different methodological approaches do not necessarily contradict one another but rather provide complementary insights at different levels of inquiry. Mendelian randomization analysis provides strong evidence for a causal relationship, LDSC analysis highlights the importance of environmental factors, and PLASCO analysis underscores the complexity of multifactorial influences. Therefore, comprehending the intricacies and mechanisms underpinning the association between the CCR and osteoporosis necessitates further dedicated research to unravel the intricate interplay among genetics, environment, and potential causality.

The current study has its strengths and limitations. The principal strength of this study lies in its deployment of the prospective cohort design sourced from the UK Biobank, further bolstered by its large sample size. Based on the results of observational studies, we performed MR analysis to investigate whether the causal association between CCR and osteoporosis/fracture is genetically determined. Nevertheless, several limitations in this study should be acknowledged and considered. First, it is imperative to recognize that the study’s exclusive focus on European populations may impede the generalizability of its findings to Asian populations due to potential inter-ethnic genetic variations. Second, while we meticulously adjusted for a spectrum of confounding factors in our analyses, the possibility of unmeasured or unidentified confounders cannot be entirely ruled out. Third, it is essential to acknowledge the inherent challenge of estimating false positive rates when employing various analytical methods, and this remains an intricate aspect to calculate accurately.

In conclusion, our prospective population-based cohort study has provided compelling evidence of significant associations between serum CCR and the risk of osteoporosis and fractures. Additionally, we observed a noteworthy negative association between the CCR and the occurrence of osteoporosis or fractures. Our Mendelian randomization (MR) analysis further supports a causal relationship between the CCR and the development of osteoporosis/fracture. Taken together, these findings strongly suggest that the CCR holds promise as a potential biomarker for assessing the risk of osteoporosis and fracture.

## Data availability statement

The original contributions presented in the study are included in the article/**Supplementary Materials**, further inquiries can be directed to the corresponding authors.

## Ethics statement

The UK Biobank study was approved by the Ethical Committee of North West Multi-center Research (11/NW/0382). Each participant signed a written informed consent document. The studies were conducted in accordance with the local legislation and institutional requirements. The human samples used in this study were acquired from primarily isolated as part of your previous study for which ethical approval was obtained. Written informed consent for participation was not required from the participants or the participants’ legal guardians/next of kin in accordance with the national legislation and institutional requirements.

## Author contributions

PH: Writing – review & editing, Writing – original draft, Methodology, Formal analysis, Data curation, Conceptualization. YY: Writing – original draft, Formal analysis. HW: Writing – review & editing, Formal analysis. YZ: Writing – review & editing, Formal analysis. YG: Writing – review & editing, Formal analysis. CH: Writing – review & editing, Formal analysis. LB: Writing – review & editing. FD: Writing – review & editing. SL: Writing – review & editing, Project administration, Methodology, Conceptualization.

## Supplementary material

Supplementary Figure 1 GO analysis of pleiotropic genes. Biological processes and molecular functions enriched by pleiotropic genes associated with CCR and fracture.

Supplementary Figure 2 A schematic diagram illustrating the association between CCR and osteoporosis/fracture.
