# Supplementary figures and images for: Muscle-origin creatinine-cystatin C ratio is an osteoporosis marker in individuals with normal renal function: evidence from observational and Mendelian randomization analysis
Source: Front Endocrinol (Lausanne). 2024 May 21;15:1325320. doi: 10.3389/fendo.2024.1325320 (PMC11148261; doi:10.3389/fendo.2024.1325320)

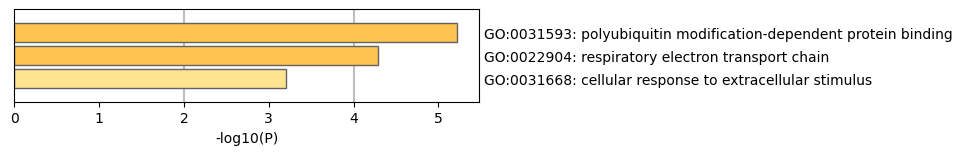

Supplement: Supplementary Figure 1 — GO analysis of pleiotropic genes. Biological processes and molecular functions enriched by pleiotropic genes associated with CCR and fracture. [file Image_1.tif]

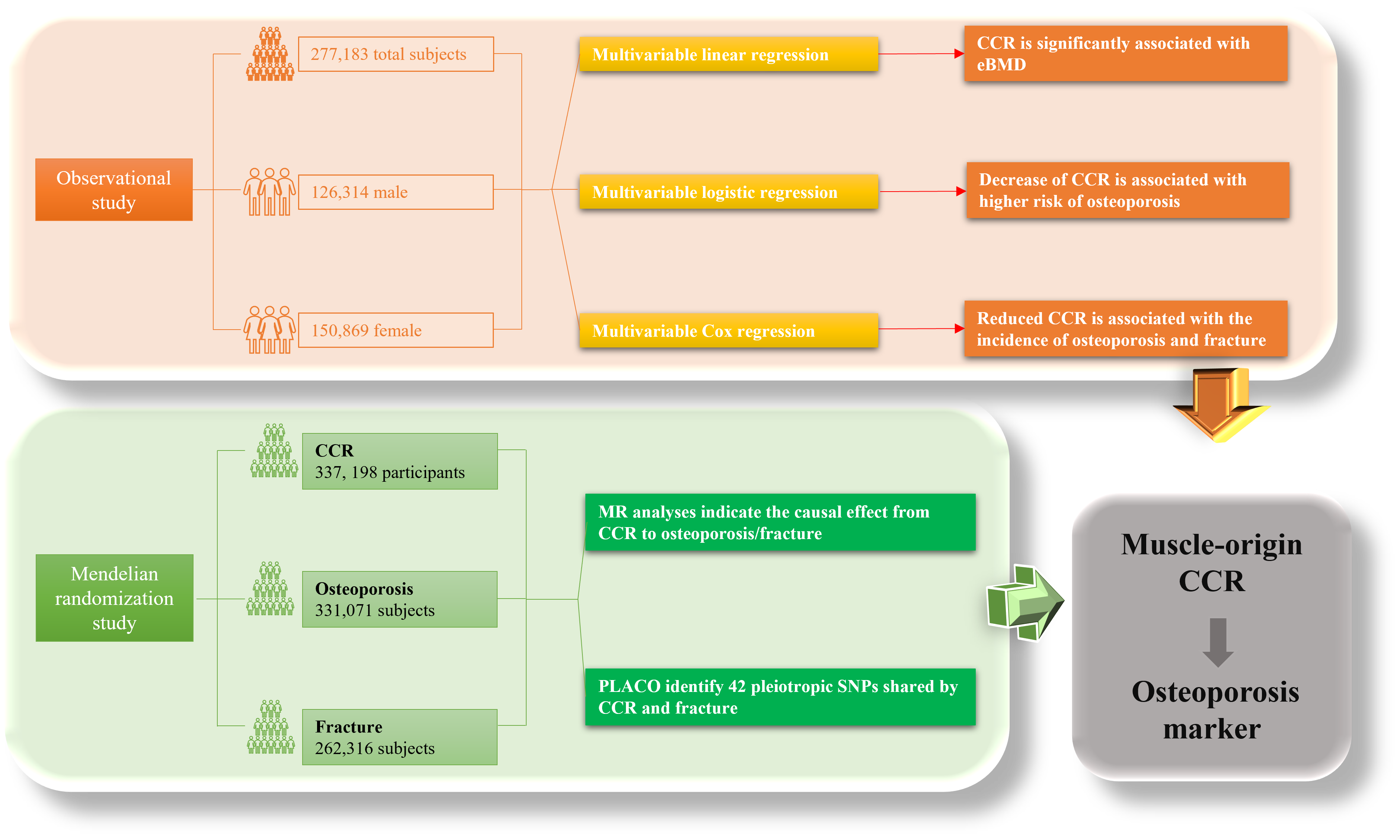

Supplement: Supplementary Figure 2 — A schematic diagram illustrating the association between CCR and osteoporosis/fracture. [file Image_2.tif]
